# The Urgency to Mitigate the Spread of Hepatitis C in Pakistan Through Blood Transfusion Reform

**DOI:** 10.15171/ijhpm.2017.120

**Published:** 2017-10-17

**Authors:** Kamiar Alaei, Mohammad Sarwar, Arash Alaei

**Affiliations:** ^1^Department of Public Administration and Policy, Rockefeller College, University at Albany, Albany, NY, USA.; ^2^Global Institute for Health and Human Rights (GIHHR), University at Albany, Albany, NY, USA.; ^3^Department of Health Policy, Management and Behavior, School of Public Health, University at Albany, Albany, NY, USA.

**Keywords:** Pakistan, HCV, Blood Transfusion Safety, Healthcare, HIV

## Abstract

Blood transfusions are contributing to a higher rate of hepatitis C virus (HCV) in Pakistan. Half of all blood transfusions in Pakistan are not screened for hepatitis C, hepatitis B or HIV. Family members donate blood that is likely not tested due to social stigma attached to HCV. Paid donations are also quite common in the country, especially by people who inject drugs (PWID), which increases the population’s exposure to HCV. Most of the population utilizes the private sector for their health needs; this sector has lax regulation due to the lack of oversight by the government or any other regulatory body. In addition, groups who are at most need for blood transfusions, such as hemophiliacs and those with thalassemia, have a higher rate of hepatitis C. This fact reinforces the need for blood transfusion reform in Pakistan, which includes improving oversight, upgrading infrastructure and promoting health literacy through cultural norms, according to the World Health Organization (WHO) recommendations. The lessons learned in Pakistan can be adapted to countries facing similar issues.

While some bloodborne diseases such as HIV/AIDS have been given serious attention in Pakistan, hepatitis C virus (HCV) has received inadequate effort and still remains a serious public health issue. There are 240 000^1^ new HCV cases in the country yearly and these have been attributed largely to medical procedures,^2^ even the death of renowned devotional singer Nusrat Fateh Ali Khan was blamed on infected medical equipment that caused him to contract HCV.^3^ Blood transfusion practices in particular are now being found to contribute to a high rate of hepatitis C in Pakistan; transfusions which are meant to save lives, are now putting people at a greater risk of exposure to HCV. In a developing country like Pakistan, unsafe blood transfusions could cause a serious socioeconomic impact in the long run and it is not the only nation that is facing this concern. Examining the case of Pakistan would be relevant to other countries dealing with similar structural and cultural barriers in order to overcome blood transfusion safety obstacles, and to prevent the outbreak of other potential epidemics, such as HIV and hepatitis B virus (HBV).

It has been estimated that up to 40% of transfused blood in Pakistan is not screened for any communicable diseases in general.^4^ Particularly, about half of blood transfusions are not screened for hepatitis C, hepatitis B or HIV.^5^ These statistics are concerning, especially when considering the high level of hepatitis C infections among those who receive regular blood transfusions. In fact, the percentage of hepatitis C among patients with thalassemia and hemophilia is estimated to be as high as 56% and 20%, respectively.^6^ The patients who are infected with these diseases must often receive regular transfusions as a part of therapy, which makes their high HCV rates particularly relevant. Compounding this is another study of patients with hepatitis C; over 48% of these patients had a history of blood transfusions in the past 6 months as the main risk factor for HCV.^7^ It is worth considering that an even greater number of people could be infected through the reuse of syringes or use of blood belonging to the initially infected recipients. Therefore, given these facts it is very important to investigate the contributing elements of hazardous blood transfusions.

The World Health Organization (WHO) has laid out guidelines to ensure that blood transfusions are performed in a safe, sanitary manner.^8^ Pakistan is dealing with challenges in several of these categories, such as *implementing a policy on blood screening that defines national requirements*, *screening all blood donations for a variety of infections including HCV*, *adequate resources being made available for screening*, *properly training enough staff* and *regulatory systems being established to oversee all public and private blood transfusions*, among others which may explain its high rate of HCV infection through blood transfusions. There are three main areas of concern in the country, which pertain to oversight, infrastructure and culture.

Pakistan has been seen to have an inadequate system of oversight, especially in the medical field.^9^ In fact, it’s national health ministry was dissolved for a number of years, preventing the formulation of a decisive countrywide health plan. The regulation of the entire medical sector is very lax, and devolution of healthcare responsibilities to the provincial governments have made implementation of reforms ever more difficult.^10^ National guidelines on the clinical use of blood were established in 1999 but have not been implemented to date,^11^ though most government clinics do attempt to screen for hepatitis C and B, and HIV.^12^ The healthcare system of Pakistan consists of a public three tiered system; at the federal, provincial and district levels. The federal government is responsible for policy development, provincial governments must provide healthcare services, and district governments are responsible for actual service delivery through primary and secondary health facilities.^13^ The aforementioned devolution in the healthcare sector has given more responsibility for improved service delivery to district facilities. These health facilities are meant to be free for the public, but they may suffer from mismanagement or a lack of resources^14^; in fact only 1% of national gross domestic product (GDP) is spent on healthcare by the government, one of the lowest in the region.^15^ This decreases their effectiveness in providing effective services. Therefore, much of the population opts to use the private sector for its health care needs, which provides upwards of 70% of the healthcare provision in Pakistan.^16^ Contrary to the government clinics, the private sector sees little regulation and many of its medical procedures go unregistered.

The blood transfusion system in Pakistan lacks uniformity and consists of 1830 blood centers, of which 85% are private entities or non-governmental organizations (NGOs) and many of the rest are public clinics.^11^ The system sees very low rates of voluntary blood donation; only about 13% of blood donations in the country come from voluntary donations, with much of the rest being obtained from other sources, such as family donations.^17^ There are estimated to be around 1.5 million documented blood transfusions in Pakistan yearly, with many patients receiving multiple transfusions.^18^ The number of undocumented blood transfusions could be far greater due to the informal nature of the Pakistani health system, which means enforcing regulations to maintain the integrity of transfusions is a challenge. This is a serious problem given the gaps that are present in the surveillance system for transfusions, and that as much as a third of blood donors have hepatitis C.^19^ Paid blood donations can also be complicit in the spread of HCV when there is a lack of proper screening; one study found that 31% of blood transfusion transactions used blood from paid donors.^20^ Many people who inject drugs (PWID) sell blood to earn a living and considering that as much as 94% of the PWID population in parts of Pakistan may be infected with HCV, this is particularly concerning. Exacerbating this concern is the possibility that many of these transfusions may go untested.^21^

The lack of infrastructure and scarcity of human resources also affect the safety of blood transfusions. For instance in Larkana, a district dealing with many HCV cases, there is a shortage of doctors who are HCV specialists, and clinics in the area lack adequate blood banking practices.^22^ Although Larkana is relatively undeveloped, there is little to suggest that the situation in more developed parts of the country is significantly different. As of 2013, there was no governmental body to regulate blood transfusions in even the most developed province in the country, Punjab.^8^ Blood centers across the country lack temperature monitored equipment to store test kits properly, and more than half of blood centers lack an internal quality audit system or even an algorithm that screens for transfusion transmissible infections.^23^ Difficulties such as the lack of a dependable power supply also prevent clinics from using the proper equipment to maintain hygiene during transfusions, or from keeping donated blood at the proper temperature.^8^

In addition to structural barriers, there are cultural barriers. There is still a certain stigma attached to being HCV positive; it often affects marriages, relationships with family and may even cause depression among patients.^24^ Consequently, people are not willing to talk about HCV, brushing it under the rug instead. Complicating this issue even further is the fact that blood is often donated to patients from relatives, who are unwilling to state their hepatitis C status due to the fear of being shamed, or of losing a family member due to the lack of blood.^25^

The government of Pakistan attempted to address the hepatitis C epidemic through the Prime Minister’s Program for the prevention and control of hepatitis viral infection.^26^ This was an unsuccessful 5-year program that ended in 2010; there were a few thousand patients who were treated for hepatitis infections, but with poor follow up and little systemic change its effectiveness was stymied. There was no effort to put in the necessary infrastructure to enforce regulations and blood transfusion safety. After this, there has been a call for institutional and legislative reform in the Pakistani health sector, but little has changed.^10^

With an epidemic of this scale, treatment of the multitude of infected is difficult, so the cure must be prevention. Among many steps, at least four should be prioritized to reduce the prevalence of this disease: (1) To create a national taskforce that implements the National Guidelines for blood transfusions that were established; (2) Alleviate infrastructural issues by providing clinics with proper equipment (ie, testing algorithms, generators) to ensure a sanitary environment for blood transfusions and by training adequate medical staff in HCV prevention and blood transfusion safety; (3) Document and monitor clinics (public and private) across the country to ensure compliance with blood screening policies; (4) Use media and cultural outlets to spread awareness about safe transfusions and recruit voluntary blood donors to ensure a safe and continuous blood donor pool. Implementing these strategies in Pakistan would allow policymakers in order to have a better understanding of how more effective approaches to overcome blood transfusion safety barriers can be developed. The lessons that are learned can then be adapted to prevent the spread of other diseases, such as HIV.

## Acknowledgements

The Global Institute for Health and Human Rights (GIHHR), University at Albany, Albany, NY, USA supported the Research Assistant (Mohammad Sarwar) in funding his research for this project.

## Ethical issues

Not applicable.

## Competing interests

Authors declare that they have no competing interests.

## Authors’ contributions

MS performed the literature review and wrote the draft. KA provided research guidance and AA gave comments and reviewed the draft.

## Authors’ affiliations

^1^Department of Public Administration and Policy, Rockefeller College, University at Albany, Albany, NY, USA. ^2^Global Institute for Health and Human Rights (GIHHR), University at Albany, Albany, NY, USA. ^3^Department of Health Policy, Management and Behavior, School of Public Health, University at Albany, Albany, NY, USA.
